# Deep Learning to Predict EGFR Mutation and PD-L1 Expression Status in Non-Small-Cell Lung Cancer on Computed Tomography Images

**DOI:** 10.1155/2021/5499385

**Published:** 2021-12-31

**Authors:** Chengdi Wang, Xiuyuan Xu, Jun Shao, Kai Zhou, Kefu Zhao, Yanqi He, Jingwei Li, Jixiang Guo, Zhang Yi, Weimin Li

**Affiliations:** ^1^Department of Respiratory and Critical Care Medicine, West China Hospital, West China Medical School, Sichuan University, Chengdu, China; ^2^Machine Intelligence Laboratory, College of Computer Science, Sichuan University, Chengdu, China

## Abstract

**Objective:**

The detection of epidermal growth factor receptor (EGFR) mutation and programmed death ligand-1 (PD-L1) expression status is crucial to determine the treatment strategies for patients with non-small-cell lung cancer (NSCLC). Recently, the rapid development of radiomics including but not limited to deep learning techniques has indicated the potential role of medical images in the diagnosis and treatment of diseases.

**Methods:**

Eligible patients diagnosed/treated at the West China Hospital of Sichuan University from January 2013 to April 2019 were identified retrospectively. The preoperative CT images were obtained, as well as the gene status regarding EGFR mutation and PD-L1 expression. Tumor region of interest (ROI) was delineated manually by experienced respiratory specialists. We used 3D convolutional neural network (CNN) with ROI information as input to construct a classification model and established a prognostic model combining deep learning features and clinical features to stratify survival risk of lung cancer patients.

**Results:**

The whole cohort (*N* = 1262) was divided into a training set (*N* = 882, 70%), validation set (*N* = 125, 10%), and test set (*N* = 255, 20%). We used a 3D convolutional neural network (CNN) to construct a prediction model, with AUCs of 0.96 (95% CI: 0.94–0.98), 0.80 (95% CI: 0.72–0.88), and 0.73 (95% CI: 0.63–0.83) in the training, validation, and test cohorts, respectively. The combined prognostic model showed a good performance on survival prediction in NSCLC patients (C-index: 0.71).

**Conclusion:**

In this study, a noninvasive and effective model was proposed to predict EGFR mutation and PD-L1 expression status as a clinical decision support tool. Additionally, the combination of deep learning features with clinical features demonstrated great stratification capabilities in the prognostic model. Our team would continue to explore the application of imaging markers for treatment selection of lung cancer patients.

## 1. Introduction

Lung cancer is the leading cause of cancer-related deaths and the second most commonly diagnosed cancer around the world, with around 1.8 million deaths and 2.2 million new cancer cases in 2020 [1]. Non-small-cell lung cancer (NSCLC) is the most common subtype of lung cancer, and the 5-year survival rate is less than 20%. The emergence of targeted therapy and immunotherapy has revolutionized the treatment of lung cancer and improved clinical outcomes among a subset of patients [2, 3]. Tyrosine kinase inhibitors (TKIs) targeted to epidermal growth factor receptor (EGFR) could lead to extend progression-free survival (PFS) compared with conventional chemotherapy in EGFR-mutated NSCLC patients [4, 5]. Simultaneously, immune checkpoint inhibitors (ICIs) targeted to the programmed death ligand-1 (PD-L1) expressed by tumor cells would also contribute to prolonged overall survival (OS) in PD-L1-positive patients with advanced NSCLC [6, 7]. Therefore, it is extremely essential to identify the genetic status of patients in the era of precision medicine.

At present, molecular genetic testing based on tumor tissue specimens is the gold standard to determine the genetic status. However, the common methods to obtain these tissue specimens, such as surgery or biopsy, are invasive, expensive, and slow, and tumor tissue varies in regard to time and space. In addition, other limitations including, but not limited to, the difficulty to obtain materials, the potential requirement for a secondary biopsy, and poor DNA quality can delay subsequent treatment decisions [8, 9]. Therefore, a noninvasive, convenient, and efficient method to predict genetic status is of imminent need.

As an effective screening tool of lung cancer, computed tomography (CT) can effectively reduce the mortality of lung cancer with early detection and is, thus, widely used in clinical examinations [10, 11]. In the past decade, radiomic methods, especially the deep learning technology, have unearthed high-throughput information in medical images [12]. Deep learning achieved a favorable performance in detecting lymph node metastases in breast cancer and estimating malignancy risk in lung cancer and diagnosing quickly in the COVID-19 pandemic [13–15]. Previous studies have shown that the features extracted from CT images of lung cancer cases might be related to gene expression patterns [16, 17]. Wang et al. used an end-to-end deep learning model to dissect CT images and to predict EGFR mutation status [18]. Tian et al. provided a deep learning model to predict high PD-L1 expression of NSCLC and to infer clinical outcomes in response to immunotherapy [19]. Based on these former explorations, to better satisfy the needs of clinical practice, there still is need for explorations of multigene expression using deep learning techniques.

Herein, we proposed a new approach to predict EGFR mutation and PD-L1 expression status in NSCLC patients based on deep learning technology and selected features to build a prognostic model. This noninvasive and easy-to-use method would assist clinicians in making treatment decisions for patients.

## 2. Materials and Methods

### 2.1. Data Acquisition and Processing

Eligible patients diagnosed/treated at the West China Hospital of Sichuan University from January 2013 to April 2019 were identified retrospectively. The inclusion criteria of patients were as follows: (1) pathologically diagnosed with primary NSCLC; (2) tested EGFR mutation and PD-L1 expression status; and (3) available CT images within 1 month before pathological diagnosis. The exclusion criteria of patients were as follows: (1) missing critical clinical data; (2) without genetic testing or having failed in the tests with poor tissue quality; and (3) without chest CT examination, or with CT images where the lesion was hard to distinguish and annotate, like being adhered to the hilar or caused atelectasis.

In total, 1262 patients were collected for this study and divided into a training set (*N* = 882), validation set (*N* = 125), and test set (*N* = 255) with a ratio of 7 : 1 : 2. Then demographic information (age, sex, and smoking history), histopathology reports, therapy (targeted therapy, ICIs), and gene testing reports were collected from the hospital information system. Thin-layer (1–3 mm) CT scanning images from multiple scanners (GE, Philips, Siemens United Imaging Health) were collected. Our follow-ups for all patients ended in April 2021. Ethics approval was obtained from the ethics committee of West China Hospital, Sichuan University.

We collected tumor specimens through biopsy or surgical resection. Then, EGFR mutation status was determined by Amplification Refractory Mutation System-Polymerase Chain Reaction (ARMS-PCR) or next-generation sequencing (NGS). PD-L1 expression status was detected using SP142 antibody in immunohistochemical (IHC) assays performed on the Ventana Benchmark platform. After being reviewed by senior pathologists, the testing results of these genes were regarded as the gold criteria in the current study.

### 2.2. Development of the Deep Learning Model

The chest CT images were taken with standard parameters and stored in DICOM format. Tumor region of interest (ROI) was delineated manually by experienced respiratory medicine specialists and then adjusted to 48 × 224 × 224 pixels from original lung window images with the original centers. The details of the adjustment were as follows: if the original scale of ROI was larger than 48 × 224 × 224 pixels, the exceeding part was cut; by contrast, if the original scale of ROI was less than 48 × 224 × 224 pixels, baseline values would be filled to standardize the size of the region. These 48 × 224 × 224 ROIs were then used to develop our deep learning model, during which they were divided into training, validation, and test sets with a ratio of 7 : 1 : 2 counting by individual patient. In regard to the genetic features, ROIs were categorized into four categories: double-negative, EGFR(−) but PD-L1(+), EGFR(+) but PD-L1(−), and double-positive.

**Figure 1:**
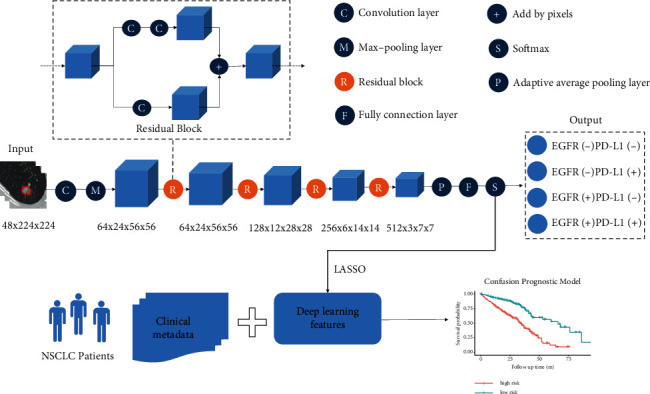
The framework of deep learning model for gene mutation classification and prognosis prediction. The deep learning model is composed of 3D convolutional neural network (CNN) for classifying the EGFR and PD-L1 expression status, and the prognostic model based on clinical metadata and deep learning features was also implemented.

As the previous literature suggested, residual block could relieve the gradient disappearance problem caused by the depth of neural network, and three-dimensional residual network showed a good performance on not only natural images [20] but also medical images [21–24]. In the current study, considering the format of CT scans, we constructed a 3D convolutional neural network (CNN) model for classifying the EGFR and PD-L1 status. In Figure 1, the architecture of our CNN network is shown. More details of layers were presented in Supplement materials (Table S1 and Table S2). Additionally, the Gradient-weighted class activation mapping (CAM) was utilized to localize and visualize the important regions in the input images for predicting the target concept.

During the training process, the batch size of every training epoch was 16. Also, the model with the best performance on the validation dataset was selected for further testing.

### 2.3. Development of the Prognostic Model

The CNN extracted 512-dimensional deep learning features of patients in the training set. Next, the least absolute shrinkage and selection operator (LASSO) method, commonly applicable for the regression of high-dimensional data [25], was used based on glmnet package. We used the “multinomial” option to adapt to multiclass datasets and changed the value of the regularization parameter lambda to adjust the LASSO model. In order to prevent overfitting, 5-fold cross validation was used to resample the training set. The model with the smallest misclassification error was selected as the optimal model, which contained the best feature set.

Then, a prognostic model combining deep learning (DL) features and clinical features (age, sex, smoking history, EGFR-TKI targeted therapy, and ICI therapy) was generated to divide the patients into high-risk and low-risk groups according to the cutoff value based on survminer package. The performance of this model was evaluated in the validation set and the test set. In addition, we also constructed a clinical prognostic model for comparison in regard to sex, age, and smoking, with targeted therapy and ICI therapy.

### 2.4. Statistical Analysis

The ANOVA test and chi-square test were used to evaluate the difference between continuous variables and categorical variables in the basic data, respectively. CNN, one of the most important deep learning models, was used to construct the prediction model. In developing the prognostic model, we reduced dimensions by using LASSO and compare variables in Cox proportional hazards regression and the log-rank-test. Tumor ROI was outlined with ITK-SNAP software. All analyses were conducted with R 4.0.2 (R Foundation for Statistical Computing, Vienna, Austria) and Python 3.10 (Python Software Foundation). Two-sided *p* values of <0.05 were regarded as statistically significant.

## 3. Results

### 3.1. Clinical Characteristics

The clinical characteristics of 1262 patients are shown in Table 1. The median age at diagnosis was 57.70 ± 10.50 years. 49.13% of patients were male. 59.35% of patients never smoked. The numbers of people in the four gene expression groups of double-negative, EGFR(−) but PD-L1(+), EGFR(+) but PD-L1(−), and double-positive were 276 (1.87%), 290 (22.98%), 502 (39.78%), and 194 (15.37%), respectively. As for the novel treatment strategies, 391 patients in the dataset had EGFR-TKI-targeted treatment while 15 received ICI. 41.91% of patients were diagnosed as stage I. The median follow-up was 31 (95%CI: 30–31) months, and the median overall survival (OS) was 44 (95%CI: 42–49) months. There was no significant difference among the training, validation, and test cohorts regarding age (*p*=0.95), sex (*p*=0.35), smoking (*p*=0.18), gene mutation status (*p*=01.00), EGFR-TKI-targeted therapy (*p*=0.39), ICI therapy (*p*=0.42), histopathology (*p*=0.82), tumor stage (*p*=0.22), median follow-up time (*p*=0.09), and overall survival (*p*=0.20).

### 3.2. Prediction Model Performance

**Figure 2:**
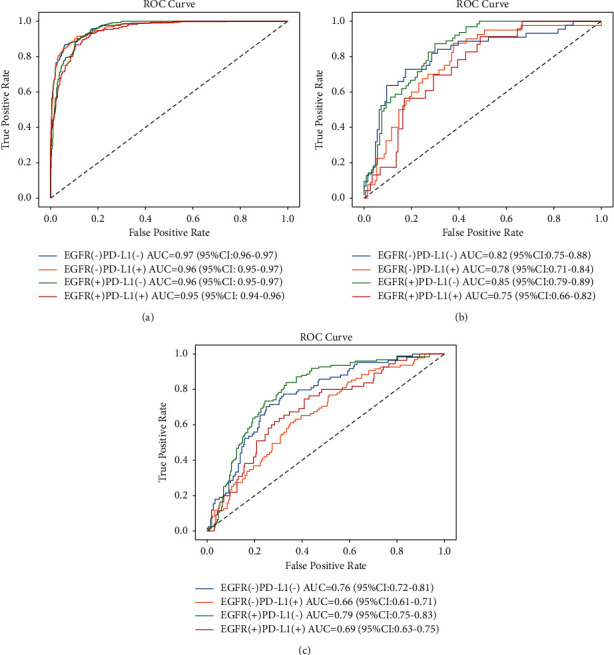
Performance of the deep learning model for the prediction of EGFR and PD-L1 expression status in training (a), validation (b), and test (c) sets by receiver operating characteristic (ROC) curves.

**Figure 3:**
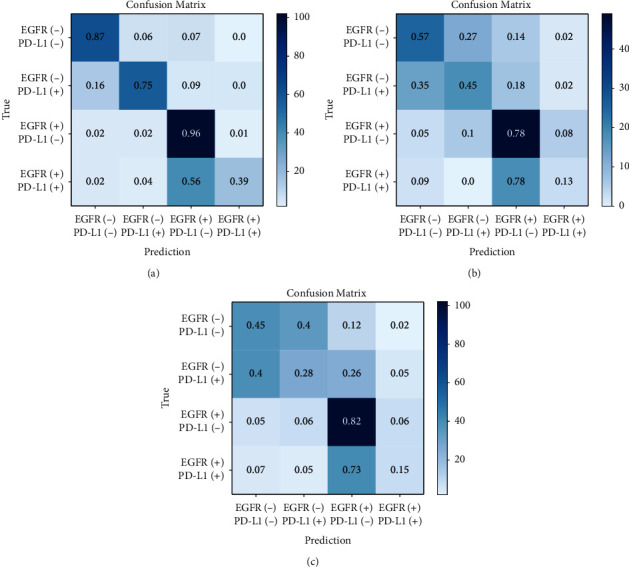
Confusion matrix of prediction model in training (a), validation (b), and test cohorts (c), respectively. The micro-average accuracies (ACCs) were 0.90 (95% CI: 0.86–0.93), 0.78 (95% CI: 0.70–0.86), and 0.75 (95% CI: 0.65–0.85) in the training, validation, and test cohorts, respectively.

**Figure 4:**
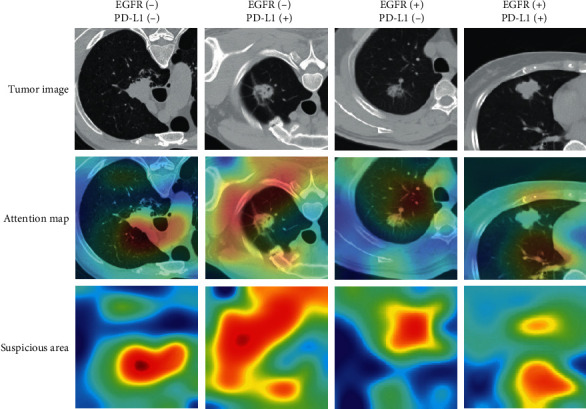
Attention map of suspicious tumor area using CAM. Suspicious tumor areas were indicated according to the attention map of the deep learning model.

Table 2 lists the predictive performance of the deep learning model evaluated with area under the ROC curve (AUC), accuracy, sensitivity, and specificity in training, validation, and test sets. The macro-average AUCs were 0.96 (95% CI: 0.94–0.98), 0.80 (95% CI: 0.72–0.88), and 0.73 (95% CI: 0.63–0.83) in the training, validation, and test cohorts, respectively. AUC of either gene classification was greater than 0.95 in the training set and greater than 0.65 in the test set (Figure 2). Our prediction system achieved an accuracy of 0.90 (95% CI: 0.86–0.93), a sensitivity of 0.74 (95% CI: 0.31–1), and a specificity of 0.93 (95% CI: 0.79–1) in the training set for the overall four-way classification. In Figure 3, as the confusion matrix of different datasets showed, most errors occurred in the adjacent groups. The deep learning model generated an attention map through CAM indicating the importance of each part in the tumor, and the dark areas might be the tissue between the tumor and the hilum (Figure 4).

### 3.3. Prognostic Model Performance

**Figure 5:**
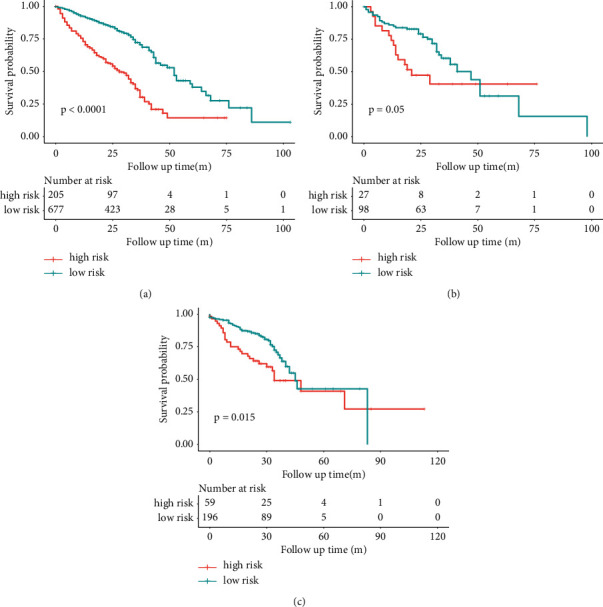
Kaplan–Meier curves in the high-risk and low-risk groups stratified by confusion prognostic prediction model in training (a), validation (b), and test sets (c). When the patients were stratified into high-risk and low-risk groups, Kaplan–Meier curves of progression to poor prognosis showed a distinct difference in survival probability in this cohort.

We built a clinical prognostic model based on several clinical features (Table S3). The C-index was 0.64 (95%CI:0.60–0.68). Then, we combined the 8 deep learning features from the softmax layer with the clinical features to build a new prognostic model, with a C-index of 0.71 (95%CI: 0.68–0.74). This prognostic model successfully stratified patients into high-risk and low-risk groups in regard to the risk of poor prognosis (death) (Figure 5). There was a significant difference in the overall survival (OS) between these groups (*p* < 0.05 both in training and test sets).

## 4. Discussion

Rapid determination of gene mutation status is crucial for the therapy decision, especially for patients who are potentially suitable for EGFR-TKI or ICI treatment. In this study, a rapid approach using deep learning based on CT images was proposed to predict EGFR mutation and PD-L1 expression status in NSCLC, with AUCs of 0.96, 0.76, and 0.76 in the training, validation, and test cohorts. Patients with positive mutation might be likely to benefit from TKI and/or ICI treatments, while patients with double-negative mutation can barely present a promising response to these treatment strategies and should adopt other therapies as soon as possible. Furthermore, the predictive model was further developed to stratify patients based on an evaluation of their risk of poor prognosis, potentially serving as a critical clinical reference.

In the field of lung cancer, radiomics has developed rapidly due to the availability of chest CT and the integration of artificial intelligence (AI) [26]. On the one hand, chest CT examination is the most routine detection method in the diagnosis and treatment process of lung cancer, since it is noninvasive, convenient, and easy to obtain in routine clinical workflow. Almost all NSCLC patients would undergo multiple CT examinations in order to track the progression of tumor lesions. On the other hand, in recent years, AI technology, especially deep learning, has been widely applied to the interpretation of medical images. Deep learning technology holds endless potential for lung cancer screening, diagnosis, and treatment, from the detection of lung nodules to identify benign and malignant lung nodules and further subtype classification [14, 27, 28].

A great deal of attention has been paid to studies that combine genomics and radiomics. In the era of precision medicine, there is a trend that patients with lung cancer are treated only after having their gene expression clarified. Some previous studies have used deep learning technology to predict EGFR, PD-L1, or ALK gene status, respectively, and achieved favorable performances (Table 3) [18, 19, 29–34]. However, these previous studies focused specifically on predicting mutation status in only one gene. Also, the current study has been the first study to predict the status of EGFR mutation and PD-L1 expression simultaneously using chest CT scans from the so-far largest cohort. At the same time, the CAM method that we utilized in this study visualized the prediction model and improved understanding of deep learning, which once was referred to as the “black box.” Another advantage of our model was that we had input 3D images, which might account for the fact that the fusion prognosis model performed better than the simple clinical model and the 3D results could fully display the characteristics of the lesion and provide more abunant image information.

More and more studies have demonstrated that image features can predict gene status and treatment response and might assist clinical practice in the future [35, 36]. Still, there were several details to be addressed. For example, when the output layer of this model was set to two categories, we got two models to predict EGFR mutation and PD-L1 expression status separately. In spite of these models' ability to achieve the research goal, they showed instability in their performance. Some studies have suggested the correlation between EGFR and PD-L1 expression [37], which may explain the stability of the four-class model in this study. Although the four categories could reflect the relationship between genes, more data from multi-centers should be needed for further improvement of model performance. Therefore, how to build a more clinically practical model will be the focus of our attention in the future.

Our research has several limitations. Firstly, it was a single-center retrospective study, but we would, to some extent, release the problem by testing the generalization and robustness of the model in an external dataset. Secondly, we temporarily lacked the assessment of the response to the treatment, which was a concern for targeted therapy and immunotherapy drugs. Thirdly, we mainly focused on two major valuable molecules: EGFR and PD-L1 are tested in the routine clinic practice. But, other genes including, but not limited to, ALK and ROS1 and gene panel are still worth investigating. If deep learning model predicts the wrong gene expression/mutation status, patients would receive the inappropriate treatment. Molecular tests are still needed to double make sure the therapy is secure before AI software will approve. Furthermore, we would try to incorporate a variety of indicators related to prognosis, such as tumor size, volume, shape, ground glass opacity (GGO), or solid components, to optimize the prognostic model in the future.

## 5. Conclusions

In conclusion, a noninvasive and effective model was proposed to predict EGFR mutation and PD-L1 expression status, which can serve as a clinical decision support tool. Additionally, the combination of deep learning features with clinical features improved stratification capabilities of the prognostic model. Later, our team will further dig deep into the application of imaging markers in the treatment decision for lung cancer patients.

## Figures and Tables

**Table 1:** Demographic and clinical characteristics of included NSCLC patients.

Characteristics	Total (*N* = 1262)	Training set (*N* = 882)	Validation set (*N* = 125)	Test set (*N* = 255)	*p* value
Age at diagnosis (year)					
Mean ± SD	57.70 ± 10.50	57.69 ± 10.27	57.48 ± 9.70	57.85 ± 10.27	0.95
Sex					
Male	620 (49.13)	438 (49.66)	66 (52.80)	116 (45.49)	0.35
Female	642 (50.87)	444 (50.34)	59 (47.20)	139 (54.51)	
Smoking					
Current/former	452 (35.82)	323 (36.62)	45 (36.00)	84 (32.94)	0.18
Never	749 (59.35)	520 (58.96)	77 (61.60)	152 (59.61)	
Unknown	61 (4.83)	39 (4.42)	3 (2.40)	19 (7.45)	
Gene mutation status					
EGFR(−) PD-L1(−)	276 (21.87)	193 (21.88)	28 (22.40)	55 (21.57)	1.00
EGFR(−) PD-L1(+)	290 (22.98)	203 (23.02)	28 (22.40)	59 (23.14)	
EGFR(+) PD-L1(−)	502 (39.78)	350 (39.68)	50 (40.00)	102 (40.00)	
EGFR(+) PD-L1(+)	194 (15.37)	136 (15.42)	19 (15.20)	39 (15.29)	
EGFR-TKI-targeted therapy					
Yes	391 (30.98)	265 (30.05)	45 (36.00)	81 (31.76)	0.39
No	871 (69.02)	617 (69.95)	80 (64.00)	174 (68.24)	
ICI therapy					
Yes	15 (1.19)	12 (1.36)	0 (0)	3 (1.18)	0.42
No	1247 (98.81)	870 (98.64)	125 (100.00)	252 (98.82)	
Histopathology					
LUAD	1185 (93.90)	824 (93.42)	119 (95.20)	242 (94.90)	0.82
LUSC	53 (4.20)	41 (4.65)	4 (3.20)	8 (3.14)	
Others	24 (1.90)	17 (1.93)	2 (1.60)	5 (1.96)	
Tumor stage					
I	529 (41.91)	378 (42.86)	43 (34.40)	108 (42.35)	0.22
II	96 (7.61)	60 (6.80)	9 (7.20)	27 (10.59)	
III	236 (18.70)	160 (18.14)	30 (24.00)	46 (18.04)	
IV	367 (29.08)	262 (29.71)	37 (29.60)	68 (26.67)	
Unknown	34 (2.69)	22 (2.49)	6 (4.80)	6 (2.35)	
Follow-up					
Median follow-up time (month, 95% CI)	31 (30–31)	30 (30–31)	31 (29–35)	32 (30,33)	0.09
Overall survival					
Death	412 (32.65)	283 (32.08)	51 (40.80)	78 (30.59)	0.20
Median OS (month, 95% CI)	44 (42–49)	43 (41–49)	41 (33-NA)	46 (40-NA)	

LUAD: lung adenocarcinoma; LUSC: lung squamous cell carcinoma; SD: standard deviation; OS: overall survival; CI: confidence interval.

**Table 2:** Predictive performance in predicting EGFR mutation and PD-L1 expression status.

	EGFR	PD-L1	ACC (95%CI)	AUC (95%CI)	Sensitivity (95%CI)	Specificity (95%CI)
Training set	−	−	0.92 (0.91–0.93)	0.97 (0.96–0.97)	0.87 (0.83–0.9)	0.93 (0.92–0.95)
	−	+	0.91 (0.89–0.93)	0.96 (0.95–0.97)	0.75 (0.7–0.78)	0.97 (0.95–0.98)
	+	−	0.87 (0.85–0.89)	0.96 (0.95–0.97)	0.96 (0.94–0.98)	0.81 (0.79–0.84)
	+	+	0.91 (0.90–0.92)	0.95 (0.94–0.96)	0.39 (0.33–0.46)	1 (0.99–1)
	Average		0.90 (0.86–0.93)	0.96 (0.94–0.98)	0.74 (0.31–1)	0.93 (0.79–1)

Validation set	−	−	0.78 (0.72–0.72)	0.82 (0.75–0.88)	0.57 (0.44–0.7)	0.85 (0.79–0.9)
	−	+	0.76 (0.71–0.71)	0.78 (0.71–0.84)	0.45 (0.33–0.57)	0.86 (0.81–0.91)
	+	−	0.74 (0.68–0.68)	0.85 (0.79–0.89)	0.78 (0.69–0.87)	0.71 (0.64–0.78)
	+	+	0.84 (0.79–0.79)	0.75 (0.66–0.82)	0.13 (0.03–0.26)	0.95 (0.92–0.98)
	Average		0.78 (0.70–0.86)	0.80 (0.72–0.88)	0.48 (0.01–0.95)	0.84 (0.66–1)

Test set	−	−	0.74 (0.7–0.7)	0.76 (0.72–0.81)	0.45 (0.36–0.54)	0.82 (0.79–0.86)
	−	+	0.68 (0.64–0.64)	0.66 (0.61–0.71)	0.28 (0.21–0.36)	0.83 (0.79–0.87)
	+	−	0.73 (0.69–0.69)	0.79 (0.75–0.83)	0.82 (0.77–0.88)	0.68 (0.63–0.73)
	+	+	0.83 (0.79–0.79)	0.69 (0.63–0.75)	0.15 (0.07–0.23)	0.95 (0.93–0.97)
	Average		0.75 (0.65–0.85)	0.73 (0.63–0.83)	0.43 (0–0.92)	0.82 (0.62–1)

Abbreviation: ACC: accuracy; AUC: area under the ROC curve.

**Table 3:** Recent representative studies using deep learning to predict gene status in lung cancer patients on CT images.

Author	Year	Design	Dataset	Training cohort	Validation cohort	Test cohort	Model	Outcome	Performance reported
Baihua Zhang	2021	Retrospective multicenter on CT	914 LUAD	638	NA	71 internal; 205 external	SE-CNN + radiomics mapping	EGFR mutation	AUC 0.910 and 0.841 in internal and external test cohorts, respectively

Wei Mu	2020	Retrospective multicenter on PET/CT	681 NSCLCs	429	187	65 external	CNN	EGFR mutation treatment response	AUC 0.86, 0.83, and 0.81 in the training, internal validation, and external test cohorts, respectively

Shuo Wang	2019	Retrospective multicenter on CT	844 LUAD	603	Five-fold cross validation; 241 independent	NA	CNN	EGFR mutation	AUC 0.85 in the primary cohort; AUC 0.81 in the independent validation cohort

Wei Zhao	2019	Retrospective multicenter on CT	616 LUAD	348	116	115 internal; 37 public	CNN 3D DenseNets	EGFR mutation	AUC 0.758 and 0.750 in the internal test set and public test set

Junfeng Xiong	2018	Retrospective single-center on CT	503 LUAD	345	158	NA	CNN	EGFR mutation	An AUC (CNN) of 0.776 and an AUC (a fusion model of CNNs and clinical features) of 0.838 in the validation set

Panwen Tian	2021	Retrospective multicenter on CT	939 NSCLCs	750	93	96	KNN	PD-L1 expression treatment response	AUC 0.78, 0.71, and 0.76 in the training, validation, and test cohorts

Ying Zhu	2020	Retrospective single-center on CT	127 LUAD	NA	Five-fold cross validation	NA	CNN 3D DenseNets	PD-L1 expression	AUC more than 0.750

Zhengbo Song	2020	Retrospective multicenter on CT	1028 NSCLCs	651	286	91	CNN 3D ResNet10	ALK fusion status Treatment response	AUC(CNN) 0.8046 and 0.7754 in the primary and validation cohorts, AUC (trained by both CT images and clinicopathological information) 0.8540 and 0.8481 in the primary and validation cohorts

LUAD: lung adenocarcinoma; NSCLC: non-small-cell lung cancer; CNN: convolutional neural network; KNN: k-nearest neighbor; NA: not applicable.

## Data Availability

The data used to support the findings of this study are available on request to the corresponding author.

## Supplementary Materials

Supplementary Materials Table 1: details of residual blocks. Table 2: details of the constructed model. Table 3: prognostic Cox models with and without the DL features.
