# Supplementary material for: Deep Learning to Predict EGFR Mutation and PD-L1 Expression Status in Non-Small-Cell Lung Cancer on Computed Tomography Images
Source: J Oncol. 2021 Dec 31;2021:5499385. doi: 10.1155/2021/5499385 (PMC8741343; doi:10.1155/2021/5499385)
Supplement: Supplementary Materials — Table 1: details of residual blocks. Table 2: details of the constructed model. Table 3: prognostic Cox models with and without the DL features. [file 5499385.f1.docx]

**Supplementary materials**

**Table S1 Detail of residual blocks**

**Table S2 Detail of constructed model**

**Table S3 Prognostic Cox models with and without the DL features**

**Table S1 Detail of residual blocks**

| **Name** | **Layer** | **Kernel** | **Stride** |
| --- | --- | --- | --- |
| **Residual Block 1** | Conv_1 | 3x3x3 | 1 |
|  | Conv_2 | 3x3x3 | 1 |
|  | Conv_R | 1x1x1 | 1 |
| **Residual Block 2** | Conv_1 | 3x3x3 | 2 |
|  | Conv_2 | 3x3x3 | 1 |
|  | Conv_R | 1x1x1 | 1 |

**Table S2 Detail of constructed model**

| **Layer** | **Kernel** | **Input** | **Output** |
| --- | --- | --- | --- |
| **Conv1** | 7x7x7 | 1x48x224x224 | 64x48x112x112 |
| **Max-pooling** | 3x3x3 | 64x48x112x112 | 64x24x56x56 |
| **Residual Block 1** | - | 64x24x56x56 | 64x24x56x56 |
| **Residual Block 2 (1)** | - | 64x24x56x56 | 128x12x28x28 |
| **Residual Block 2 (2)** | - | 128x12x28x28 | 256x6x14x14 |
| **Residual Block 2 (3)** | - | 256x6x14x14 | 512x3x7x7 |
| **Adaptive average pooling** | 3x7x7 | 512x3x7x7 | 512x1x1x1 |
| **Fully connection layer** | 512 | 512x1x1x1 | 4x1 |
| **Sigmoid** | 4 | 4x1 | 4x1 |

**Table S3 Prognostic Cox models with and without the DL features**

| **Feature** | **Combined model**  **(C-Index:0.71)** | | **Clinical model**  **(C-Index:0.64)** | |
| --- | --- | --- | --- | --- |
|  | **HR (95%CI)** | ***p*** | **HR (95%CI)** | ***p*** |
| **Age** | 1.02(1.01-1.03) | <0.01 | 1.02(1.01-1.03) | <0.01 |
| **Sex** | 0.96(0.65-1.41) | 0.82 | 0.84(0.58-1.23) | 0.37 |
| **Smoke** | 1.69(1.15-2.49) | 0.01 | 1.79(1.23-2.62) | <0.01 |
| **Smoke Unknown** | 1.68(0.96-2.96) | 0.07 | 1.9(1.09-3.31) | 0.02 |
| **Target therapy** | 1.64(1.24-2.17) | <0.01 | 1.61(1.26-2.05) | <0.01 |
| **ICI therapy** | 1.24(0.6-2.55) | 0.56 | 1.15(0.57-2.35) | 0.69 |
| **DL_feature_291** | 1.25(0.4-3.93) | 0.7 | - |  |
| **DL_feature_309** | 0.43(0.17-1.08) | 0.07 | - |  |
| **DL_feature_311** | 3.78(1.3-10.98) | 0.01 | - |  |
| **DL_feature_332** | 44.85(9.96-202.02) | <0.01 | - |  |
| **DL_feature_407** | 1.86(0.63-5.48) | 0.26 | - |  |
| **DL_feature_420** | 0.1(0.03-0.4) | <0.01 | - |  |
| **DL_feature_467** | 4.16(0.59-29.13) | 0.15 | - |  |
| **DL_feature_471** | 0.24(0.06-0.92) | 0.04 | - |  |
